# Ocean acidification alters sperm responses to egg-derived chemicals in a broadcast spawning mussel

**DOI:** 10.1098/rsbl.2022.0042

**Published:** 2022-04-06

**Authors:** Rowan A. Lymbery, Jill Brouwer, Jonathan P. Evans

**Affiliations:** Centre for Evolutionary Biology, School of Biological Sciences, University of Western Australia, Perth, WA 6009, Australia

**Keywords:** broadcast spawning, climate change, egg chemoattractants, ocean acidification, sperm chemotaxis, genetic compatibility

## Abstract

The continued emissions of anthropogenic carbon dioxide are causing progressive ocean acidification (OA). While deleterious effects of OA on biological systems are well documented in the growth of calcifying organisms, lesser studied impacts of OA include potential effects on gamete interactions that determine fertilization, which are likely to influence the many marine species that spawn gametes externally. Here, we explore the effects of OA on the signalling mechanisms that enable sperm to track egg-derived chemicals (sperm chemotaxis). We focus on the mussel *Mytilus galloprovincialis*, where sperm chemotaxis enables eggs to bias fertilization in favour of genetically compatible males. Using an experimental design based on the North Carolina II factorial breeding design, we test whether the experimental manipulation of seawater pH (comparing ambient conditions to predicted end-of-century scenarios) alters patterns of differential sperm chemotaxis. While we find no evidence that male–female gametic compatibility is impacted by OA, we do find that individual males exhibit consistent variation in how their sperm perform in lowered pH levels. This finding of individual variability in the capacity of ejaculates to respond to chemoattractants under acidified conditions suggests that climate change will exert considerable pressure on male genotypes that can withstand an increasingly hostile fertilization environment.

## 1. Introduction

The increase in atmospheric carbon dioxide (CO_2_) due to anthropogenic emissions is causing considerable chemical changes to the world's oceans. Importantly, oceans act as a store for anthropogenic CO_2_, which has altered the carbonate chemistry and pH of seawater (ocean acidification; OA) [1]. OA has the potential to impact reproduction in many marine species, particularly via effects on gametes prior to fertilization [2]. This is because most marine species are broadcast spawners, where sperm and eggs are expelled into seawater for external fertilization. Therefore, the gametes of many ocean species will be exposed directly to changes in oceanic chemistry. Accordingly, negative effects of OA have been reported for sperm motility (e.g. [3,4]) and fertilization rate (e.g. [5,6]), although findings have been mixed across studies [2,7].

Our incomplete understanding of how OA affects gametes may be a result of studies typically focussing on gametes in isolation rather than sperm–egg interactions prior to fertilization (see [2]). In broadcast spawners, successful fertilization often relies on complex biochemical processes of sperm–egg communication before contact [8], typically involving reproductive fluids expelled by females or their eggs that allow sperm to track a chemical gradient towards unfertilized eggs (see reviews by [9,10])—a phenomenon known as sperm chemotaxis [11,12]. Although the potential impacts of OA on sperm chemotaxis are largely unknown, there is some evidence from broadcast spawning invertebrates that reductions in ocean pH can have significant but complex effects on the release of egg chemicals, sperm swimming behaviour and ultimately fertilization [3,6,13–15]. More broadly, there is evidence that OA can alter other biological signalling molecules (e.g. peptides) that influence behaviours in marine species [16].

An important component of sperm–egg interactions, including sperm chemotaxis, is that there are consistent differences in their outcomes among individual males, females and male–female combinations, often promoting fertilization between gametes from genetically compatible partners [8,17]. For example, in the mussel *Mytilus galloprovincialis*, a series of studies have reported that egg-derived chemoattractants differentially affect sperm. These include differential changes in sperm physiology (e.g. the acrosome reaction and structural modifications in sperm glycans; [18]), sperm swimming behaviour, fertilization and ultimately offspring viability [19–21]. However, no studies have yet tested whether such differential patterns of sperm–egg interaction, via chemical signalling, are impacted by changes to seawater chemistry such as OA.

Here, we used *M. galloprovincialis* to provide the first test of how OA affects patterns of individual-specific sperm chemotaxis. To address this question, we set up a series of partly factorial crosses, each involving two focal males and two focal females, and conducted sperm chemotaxis trials under experimentally modified pH regimens, reflecting current levels (ambient pH) and those predicted under a high CO_2_ emissions scenario (low pH). We then determined whether patterns of sperm chemoattraction exhibited by individual males, individual females or specific combinations of males and females are impacted by OA.

## 2. Methods

### (a) Mussel collection and spawning

We collected adult *M. galloprovincialis* mussels from Woodman Point (32°14′03.6″S, 115°76′25″E) during the 2019 spawning season (June–September). We have previously found ample, well-mixed segregating genetic variation in this population, which is reflected in differential patterns of chemotaxis based on within-population genetic compatibility [21]. Mussels were induced to spawn in filtered seawater (FSW) heated to 28°C [21]. We prepared FSW by dissolving Ocean Nature Sea Salt (Aquasonic, Wauchope, NSW, Australia) in deionized water to a salinity of 35 psu. The synthetic seawater was passed through several mechanical filters (final mesh size 5 µm), a carbon filter and ultraviolet light to sterilize bacteria and remove contaminants. Gametes were collected and concentrations estimated using standard procedures (electronic supplementary material).

### (b) Seawater pH manipulation

We prepared FSW at two pH levels for experimental trials: ‘ambient’ (pH approx. 8.0; reflecting current sea surface conditions) and ‘low’ (pH approx. 7.6; reflecting end-of-century predictions under a high CO_2_ emissions scenario; [22]). The low treatment was prepared by bubbling pure, commercial grade CO_2_ through FSW. The pH change was monitored using a Handylab 100 pH meter with a Blueline 24 pH electrode (Xylem Analytics). New FSW solutions were made for each of the experimental blocks (see below), with batches for the two treatments in each block stored in sealed 10 L containers until required to minimize gas exchange with the surrounding air [23]. For each treatment batch, we measured pH on the total scale (pH_T_; [24]) and total alkalinity (electronic supplementary material). Dissolved inorganic carbon (DIC) and partial pressure of CO_2_ (pCO_2_) were calculated from the total alkalinity, temperature and pH measurements using the R package ‘seacarb’ [25].

### (c) Experimental design and sperm chemotaxis trials

**Figure 1.**
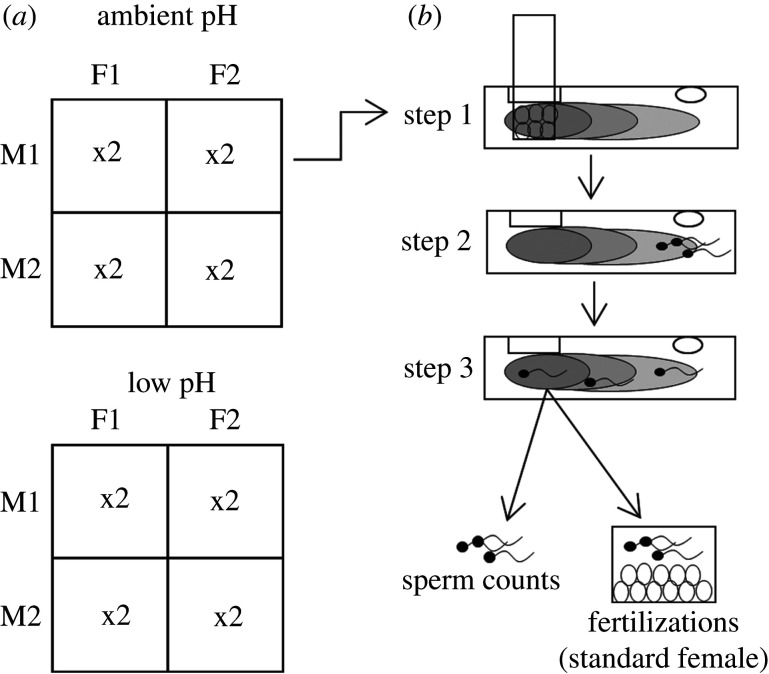
Overview of a single block of our experimental design. (*a*) Within a block, sperm from two focal males (M1 and M2) were combined with egg chemoattractants from two focal females (F1 and F2), at each pH, with every M × F × pH combination replicated twice. (*b*) Each chemotaxis trial involved establishing a gradient with focal female eggs, then the introduction of sperm to the chamber, and finally removal of sperm from centre of gradient for counts and fertilization assays (using eggs from the non-focal female).

We used a cross-classified experimental design, similar to a modified North Carolina II breeding design [26], where each experimental block consisted of two focal females and two focal males (that commenced spawning within 15 min of each other) crossed in all combinations, with two repeated measures performed per cross (figure 1*a*). Within each block, chemotaxis trials were performed separately at both pH treatments (figure 1*a*). Therefore, we could partition variance in sperm chemotaxis by fitting random effects for ‘male’ (sperm donor), ‘female’ (chemoattractant donor), ‘male × female’ (interaction between sperm and chemoattractants from different individuals) and interactions between pH and all other effects. We performed a total of 18 blocks (36 focal males, 36 focal females and 72 male–female crosses).

For each repeated measure of a male–female cross, sperm chemotaxis assays were conducted as described previously [21], using chambers made from modified 10 ml syringe tubes (electronic supplementary material). The chambers were filled with 5 ml of either ambient or low FSW. Eggs from the focal female (2 ml at 5 × 10^4^ cells ml^−1^; [21]) were suspended in a 30 µm filter mesh at one end of the chamber for 1 h to establish a chemoattractant gradient (figure 1*b*, step 1), then carefully removed. Sperm from the focal male (standardized to 1 ml at 1 × 10^8^ cells ml^−1^; enough for reliable cell counts at the final step) were added to the other end of the chamber (figure 1*b*, step 2). These concentrations generated post-chemotaxis fertilization rates (see below) that avoided floor (0%) or ceiling (100% fertilization) effects. After 10 min, a 700 µl aliquot was removed from the centre of the egg chemoattractant gradient (figure 1*b*, step 3).

Within a block, the outcome of sperm chemotaxis was assessed through two assays: (i) sperm concentration of the extracted aliquot was measured using a haemocytometer; (ii) a 250 µl subsample of the aliquot was used to fertilize eggs from a non-focal female (1 ml at 5 × 10^4^ cell ml^−1^), i.e. one female per block that was separate from the focal females (to ensure that male × female effects are attributable to focal chemoattractants; [21]) (figure 1). The first assay estimates the number of sperm accumulated, while the second incorporates both sperm accumulation and effects of chemoattractants on the physiology of sperm (e.g. extent of capacitation and acrosome reaction; [9]). Fertilization mixes were left for 2 h, then post-chemotaxis fertilization rates were assessed by counting a haphazard sample of 100 eggs and scoring the proportion undergoing polar body formation and/or cell division. Sperm counts were obtained from 18 blocks and fertilization success from 16 blocks.

As the primary aim of this study was to investigate pH effects on sperm in the chemotaxis chambers, the non-focal female eggs were prepared in ambient FSW. However, for sperm in the low pH chemotaxis trials, the addition of the 250 µl sperm sample (pH approx. 7.6) to 1 ml of the standard eggs (pH approx. 8.0) slightly lowered the overall pH during fertilizations (to pH approx. 7.9). Supplementary tests indicated that a difference in pH levels of fertilization mixes had a significant effect on fertilization rate, but this effect could not be attributed to the pre-fertilization environment and therefore we do not interpret these in the context of chemotaxis (see electronic supplementary material for full details).

### (d) Data analyses

Statistical analyses were undertaken in R v. 4.0.3 [27]. Sperm count and post-chemotaxis fertilization success were modelled with Poisson and beta-binomial generalized linear mixed models (GLMMs), respectively, using ‘lme4’ [28]. Each model included a fixed effect of pH treatment, and random effects of experimental block, male ID (sperm donor), female ID (egg chemoattractant donor) and the interactions of male × female, male × pH, female × pH, and male × female × pH. In the Poisson sperm count model, we included an observation-level random effect to account for overdispersion (electronic supplementary material). We tested significance of the fixed pH effect using Wald chi-square tests. Significance of random effects was tested by removing each effect in turn and comparing the resultant fit to full models using likelihood ratio tests.

## 3. Results

### (a) Seawater carbonate chemistry

Our experimental manipulation of seawater resulted in a mean pH_T_ (±s.e.) of 7.97 (±0.01) in the ambient treatment and 7.56 (±0.01) in the low treatment (for corresponding DIC and pCO_2_ see table 1; electronic supplementary material, table S4). The pH_T_ difference was maintained across blocks (paired *t*-test; *t*_17_ = 114.84, *p* < 0.001). There was no significant difference in total alkalinity between treatments (paired *t*-test; *t*_17_ = 0.978, *p* = 0.342; table 1). Carbonate chemistry parameters were very similar to those reported in naturally collected seawater from south-western Australia [29,30].

**Table 1.** Carbonate chemistry parameters (mean ± s.e. across 18 experimental blocks) of each FSW treatment (i.e. 36 seawater batches total, 18 in each treatment): pH on the total scale (pH_T_), total alkalinity (TA), dissolved inorganic carbon (DIC) and partial pressure of CO_2_ (pCO_2_).

treatment	pH_T_	TA (µmol kg^−1^)	DIC (µatm)	pCO_2_ (µmol kg^−1^)
ambient	7.97 ± 0.01	2421 ± 38	2272 ± 37	543 ± 41
low	7.56 ± 0.01	2416 ± 38	2396 ± 39	1517 ± 91

### (b) Sperm chemotaxis assays

#### (i) Sperm counts

There was no overall effect of pH treatment on sperm count at the centre of the chemoattractant gradient (Wald $\chi_{\;1}^{2}=0.038$, *p* = 0.846). There was significant variance in sperm counts among males, but no other significant random effects or interactions involving pH and random effects on sperm count (table 2).

**Table 2.** Results of log-likelihood ratio tests for random effects from the GLMMs of (*a*) sperm count, and (*b*) post-chemotaxis fertilization rate. *G*^2^ = –2× difference between reduced and full model log-likelihoods. AIC_c_ = Akaike information criteria with correction for finite sample sizes. Significant *p*-values are italicized.

model	log-likelihood	AIC_c_	*G^2^*	*p*
(*a*) sperm count				
full	−1461.8	2944.41		
(−block)	−1462.1	2942.94	0.68	0.411
(−male)	−1469.3	2957.33	15.07	*<0.001*
(−female)	−1461.8	2942.30	0.03	0.852
(−male × female)	−1461.9	2942.48	0.22	0.641
(−male × pH)	−1462.1	2942.87	0.61	0.434
(−female × pH)	−1461.9	2942.44	0.18	0.672
(−male × female × pH)	−1461.8	2942.26	<0.01	1.000
(*b*) fertilization rate				
full	−994.3	2009.50		
(−block)	−994.6	2007.85	0.52	0.467
(−male)	−1003.1	2024.96	17.63	*<0.001*
(−female)	−997.6	2013.90	6.57	*0*.*010*
(−male × female)	−996.5	2011.72	4.39	*0*.*036*
(−male × pH)	−997.2	2013.05	5.72	*0*.*016*
(−female × pH)	−994.68	2008.08	0.75	0.386
(−male × female × pH)	−994.30	2007.33	<0.01	1.000

#### (ii) Post-chemotaxis fertilization rates

**Figure 2.**
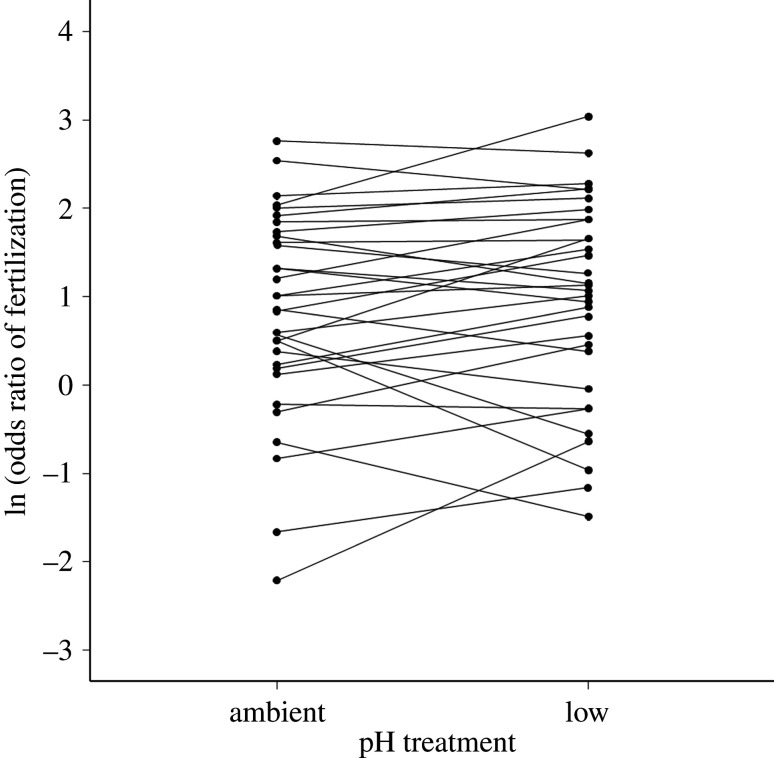
Natural logarithm of the odds ratio for fertilization rate of individual males (points and lines) at each pH treatment (i.e. pH during sperm chemotaxis).

There was no significant overall effect of pH in the chemotaxis chamber on fertilization rates (Wald $\chi_{\;1}^{2}=0.998$, *p* = 0.318). We found significant variance in fertilization rates due to male ID and female (chemoattractant) ID, and a significant interaction for male × female (table 2). There was also a significant male × pH interaction, indicating that males exhibited consistent differences in their sperm's response to pH variation (table 2 and figure 2). This effect is attributable to pH of the chemotaxis chamber, as the pH of the non-focal eggs did not affect variation among individual males (electronic supplementary material).

## 4. Discussion

Our study represents the first test of OA on individual-specific sperm chemotaxis, revealing that the effects of acidification vary depending on the spawning individuals involved. We also show that the average rate of sperm chemotaxis, and the male-by-female interactions that characterize chemotaxis and fertilization in *M. galloprovincialis* [18–20], were unaffected by pH. These latter findings suggest that compatibility-based female choice of sperm [21] can be robust to OA. However, the significant interaction between male ID and pH on post-chemotaxis fertilization rate indicates that sperm responded differently to egg chemicals at ambient and low pH, but that these changes varied in strength and direction across males. This finding could point to selective pressures on males producing sperm that are able to perform optimally in an acidified ocean.

The absence of a main effect of pH on chemotaxis (i.e. averaged across individuals) contrasts with a previous study on *M. galloprovincialis*, reporting a difference between average fertilization rates after chemotaxis in ambient and low pH conditions [15]. However, this previous study was not able to partition variation in sperm chemotaxis among different males and therefore to isolate individual-specific responses to acidification from average responses. It could be that once individual-specific effects are separated in this species, average effects of pH are relatively less important. By contrast, recent studies on sea urchins have reported both average and individual-specific effects of pH on the production of egg-associated fluids and the swimming behaviour of sperm [13,14]. These previous findings in conjunction with our current results suggest that OA might have complex and varying effects on gametes across systems, and that the full effects may not be apparent from a straightforward comparison of average gamete performance.

In addition to considering individual-specific effects of pH across focal males and females, our design incorporated the novel component of comparing male × female gametic compatibility across pH treatments. Gametic compatibility characterizes sperm–egg interactions in *M. galloprovincialis* [18–20] and other broadcast spawners [17], allowing females to bias fertilizations toward genetically compatible sperm [21]. We have previously hypothesized that OA will disrupt patterns of gamete compatibility [15], as differential chemotaxis likely depends on multifaceted chemical signals that could be sensitive to seawater chemistry. By contrast, our findings suggest that in *M. galloprovincialis*, gamete-level mate choice via chemical signals may be robust to environmental perturbance, a conclusion that also applied to temperature stress in an earlier study [31]. We recommend that future studies prioritize tests for compatibility-based gamete interactions, and their robustness to environmental change, in a range of systems to resolve the generality of these findings.

The effects of OA in our study manifested as an interaction between male ID and pH, where sperm performance improved under low pH for some males and decreased for others. Interestingly, this effect was apparent for post-chemotaxis fertilization rate but not for the number of sperm attracted, suggesting that rather than altering chemotactic movement of sperm, changes in pH alter the way that egg-derived chemicals induce changes in sperm physiology [18]. This provides a potential mechanistic explanation for previous evidence of individual-specific effects of OA on reproductive success in broadcast spawners (e.g. [32–34]). Moreover, these patterns could have important implications for the adaptation of populations under OA, as they imply that ongoing pH changes will place selective pressure on males whose sperm can maintain or improve their responses to egg signals. If the among-male variation in sperm tolerance to OA has a genetic basis, this could lead to adaptive shifts in the way sperm respond to chemoattractants. Characterizing the genetic architecture of gamete interactions, such as sperm chemotaxis, will therefore be a fruitful avenue to better understand how marine populations will respond to climate change.

## Data Availability

Data associated with this manuscript are available from the Dryad Digital Repository: https://doi.org/10.5061/dryad.9cnp5hqkf [35].

## References

[1] Caldeira K, Wickett M. 2005 Ocean model predictions of chemistry changes from carbon dioxide emissions to the atmosphere and ocean. J. Geophys. Res. **110**, C09S04. (10.1029/2004JC002671)

[2] Byrne M. 2011 Impact of ocean warming and ocean acidification on marine invertebrate life history stages: vulnerabilities and potential for persistence in a changing ocean. Oceanogr. Mar. Biol. Annu. Rev. **49**, 1-42. (10.1201/b11009-2)

[3] Morita M, Suwa R, Iguchi A, Nakamura M, Shimada K, Sakai K, Suzuki A. 2010 Ocean acidification reduces sperm flagellar motility in broadcast spawning reef invertebrates. Zygote **18**, 103-107. (10.1017/S0967199409990177) 20370935

[4] Campbell AL, Levitan DR, Hosken DJ, Lewis C. 2016 Ocean acidification changes the male fitness landscape. Sci. Rep. **6**, 31250. (10.1038/srep31250) 27531458 PMC4987666

[5] Havenhand JN, Buttler FR, Thorndyke MC, Williamson JE. 2008 Near-future levels of ocean acidification reduce fertilization success in a sea urchin. Curr. Biol. **18**, R651-R652. (10.1016/j.cub.2008.06.015) 18682203

[6] Eads AR, Kennington WJ, Evans JP. 2016 Interactive effects of ocean warming and acidification on sperm motility and fertilization in the mussel *Mytilus galloprovincialis*. Mar. Ecol. Prog. Ser. **562**, 101-111. (10.3354/meps11944)

[7] Ross PM, Parker L, O'Connor WA, Bailey EA. 2011 The impact of ocean acidification on reproduction, early development and settlement of marine organisms. Water **3**, 1005-1030. (10.3390/w3041005)

[8] Evans JP, Sherman CDH. 2013 Sexual selection and the evolution of egg-sperm interactions in broadcast-spawning invertebrates. Biol. Bull. **224**, 166-183. (10.1086/BBLv224n3p166) 23995741

[9] Kekäläinen J, Evans JP. 2018 Gamete-mediated mate choice: towards a more inclusive view of sexual selection. Proc. R. Soc. B **285**, 20180836. (10.1098/rspb.2018.0836) PMC6083266 30051836

[10] Gasparini C, Pilastro A, Evans JP. 2020 The role of female reproductive fluids in sperm competition. Phil. Trans. R. Soc. B **375**, 20200077. (10.1098/rstb2020.0077) 33070736 PMC7661459

[11] Miller RL. 1985 Sperm chemo-orientation in the metazoa. In Biology of fertilization V2: biology of sperm (eds CB Metz, A Monroy), pp. 274-337. New York, NY: Academic Press.

[12] Eisenbach M. 1999 Sperm chemotaxis. Rev. Reprod. **4**, 56-66. (10.1530/ror.0.0040056) 10051103

[13] Foo SA, Byrne M, Cristina M. 2018 Residing at low pH matters, resilience of the egg jelly coat of sea urchins living at a­ CO_2_ vent site. Mar. Biol. **165**, 97. (10.1007/s00227-018-3359-2)

[14] Foo SA, Deaker D, Byrne M. 2018 Cherchez la femme – impact of ocean acidification on the egg jelly coat and attractants for sperm. J. Exp. Biol. **221**, jeb177188. (10.1242/jeb.177188) 29674376

[15] Lymbery RA, Kennington WJ, Cornwall CE, Evans JP. 2019 Ocean acidification during prefertilization chemical communication affects sperm success. Ecol. Evol. **9**, 12 302-12 310. (10.1002/ece3.5720) PMC6854328 31832161

[16] Roggatz CC, Lorch M, Hardege JD, Benoit DM. 2016 Ocean acidification affects marine chemical communication by changing structure and function of peptide signalling molecules. Glob. Chang. Biol. **22**, 3914-3926. (10.1111/gcb.13354) 27353732

[17] Evans JP, Lymbery RA. 2020 Sexual selection after gamete release in broadcast spawning invertebrates. Phil. Trans. R. Soc. B **375**, 20200069. (10.1098/rstb.2020.0069) 33070722 PMC7661442

[18] Kekäläinen J, Evans JP. 2016 Female-induced remote regulation of sperm physiology may provide opportunities for gamete-level mate choice. Evolution **71**, 238-248. (10.1111/evo.13141) 27921298

[19] Evans JP, García-González F, Almbro M, Robinson O, Fitzpatrick JL. 2012 Assessing the potential for egg chemoattractants to mediate sexual selection in a broadcast spawning marine invertebrate. Proc. R. Soc. B **279**, 20120181. (10.1098/rspb.2012.0181) PMC3367782 22438495

[20] Oliver M, Evans JP. 2014 Chemically moderated gamete preferences predict offspring fitness in a broadcast spawning invertebrate. Proc. R. Soc. B **281**, 20140148. (10.1098/rspb.2014.0148) PMC4043089 24741014

[21] Lymbery RA, Kennington WJ, Evans JP. 2017 Egg chemoattractants moderate intraspecific sperm competition. Evol. Lett. **1**, 317-327. (10.1002/evl3.34) 30283659 PMC6121861

[22] IPCC. 2013 Climate change 2013: the physical science basis. Cambridge, UK: Cambridge University Press.

[23] Cornwall CE, Hurd CL. 2016 Experimental design in ocean acidification research: problems and solutions. ICES J. Mar. Sci. **73**, 572-581. (10.1093/icesjms/fsv118)

[24] Dickson AG, Sabine CL, Christian JR. 2007 Guide to best practices for ocean CO_2_ measurements. Sidney, BC, Canada: North Pacific Marine Science Organization.

[25] Gattuso JP, Epitalon JM, Lavigne H, Orr J. 2018 seacarb: seawater carbonate chemistry. R package version 3.2.8. (See http://CRAN.R-project.org/package=seacarb.)

[26] Lynch M, Walsh B. 1998 Genetics and analysis of quantitative traits. Sunderland, MA: Sinauer Associates.

[27] R Core Team. 2020 R: a language and environment for statistical computing. Vienna, Austria: R Foundation for Statistical Computing.

[28] Bates D, Macechler M, Bolker B, Walker S. 2015 Fitting linear mixed-effects models using lme4. J. Stat. Softw. **67**, 1-48. (10.18637/jss.v067.i01)

[29] Comeau S, Cornwall CE, McCulloch MT. 2017 Decoupling between the response of coral calcifying fluid pH and calcification to ocean acidification. Sci. Rep. **7**, 7573. (10.1038/s41598-017-08003-z) 28790423 PMC5548905

[30] Cornwall CE, Comeau S, DeCarlo TM, Moore B, D'Alexis Q, McCulloch MT. 2018 Resistance of corals and coralline algae to ocean acidification: physiological control of calcification under natural pH variability. Proc. R. Soc. B **285**, 20181168. (10.1098/rspb.2018.1168) PMC6111182 30089625

[31] Eads AR, Evans JP, Kennington WJ. 2016 Plasticity of fertilization rates under varying temperature in the broadcast spawning mussel, *Mytilus galloprovincialis*. Ecol. Evol. **6**, 6578-6585. (10.1002/ece3.2375) 27777731 PMC5058529

[32] Schlegel P, Havenhand JN, Gillings MR, Williamson JE. 2012 Individual variability in reproductive success determines winners and losers under ocean acidification: a case study with sea urchins. PLoS ONE **7**, e53118. (10.1371/journal.pone.0053118) 23300876 PMC3531373

[33] Vihtakari M, Havenhand J, Renaud PE, Hendriks IE. 2016 Variable individual- and population- level responses to ocean acidification. Front. Mar. Sci. **3**, 1-11. (10.3389/fmars.2016.00051)

[34] Smith KE, Byrne M, Deaker D, Hird CM, Nielson C, Wilson-McNeal A, Lewis C. 2019 Sea urchin reproductive performance in a changing ocean: poor males improve while good males worsen in response to ocean acidification. Proc. R. Soc. B **286**, 20190785. (10.1098/rspb.2019.0785) PMC6661356 31337311

[35] Lymbery RA, Brouwer J, Evans JP. 2022 Data from: Ocean acidification alters sperm responses to egg-derived chemicals in a broadcast spawning mussel. *Dryad Digital Repository*. (10.5061/dryad.9cnp5hqkf) PMC8984365 35382588

